# Soda Springs at Manitou

**Published:** 1874-03

**Authors:** 


### SODA SPRINGS AT MANITOU,

SITUATED on the line of the Denver and Rio Grande Railway, seventy-five miles south of Denver City, are highly estimated for their virtue in the cure of rheumatism and all cutaneous disorders, and the special class for which the practitioner's sole dependence has hitherto been mercury.

In the upper part of a rock, which had apparently been formed by deposition, is a beautiful white basin, overhung by currant bushes, in which the cold, clear water bubbles up, kept in constant motion by the escaping gas, and overflowing the rock, which it has almost entirely covered with a smooth crust of glistening white. Professor Hayden, in his report on Colorado, says:

"Perhaps the feature of the greatest general interest in this region is the Soda Springs, which are located about three miles above Colorado City, in the valley of Fountain Creek. The scenery around them is grand beyond any I have ever seen in the vicinity of any other medicinal spring. There are four of them. The first one is close to the road, within fifty feet of the creek. For a distance of sixty feet or more around the spring there is a deposit or incrustation in thin layers. About one hundred yards above the first spring is the second one, on the right side of the creek. This is much the largest one, and has formed a basin six or eight feet across, from the centre of which boils up a violent current. On the opposite side of the creek, not more than twenty-five feet from it, and located about ten feet above it, is a third small spring. The water is stronger than that of the others, and is used principally for drinking purposes. The fourth spring is perhaps fifty feet above the second, on the right side of the creek, and within four feet of the water's edge. Its waters are rather chalybeate than otherwise. The basin of the second spring is about four feet deep, and is used for bathing. The first three springs are strongly impregnated with carbonic acid gas, and are the true springs. The temperature of the springs is about 60°."

The chief spring, christened by Professor Hayden the "DOCTOR," and which is probably the richest mineral spring in the world, containing as it does over an ounce of medicated matter to every four gallons of water, will be called the "DOCTOR," or "GALEN SPRING." The Chalybeate Spring, whose waters resemble those of Pyrmont, in Europe, will hereafter be known as the "IRON UTE." The "Great Spring" will keep its present name. It will be called in plain English the "BOILING FOUNTAIN." Another, called by the Indians the "Beast," from the fact that wild beasts were wont to drink the water to heal their diseases, will be known as the "NAVAJOE."

The springs proper have been called "MANITOU," and are distant five miles from Colorado Springs, a station on the Denver and Rio Grand Railway. A good carriage road will be constructed from the station to Manitou, and a post-office and telegraph station built. Manitou lies in the celebrated Ute Pass, from which El Paso County derives its name. The natural scenery from this point is very grand. In the background, and in the centre of the semicircle, rises the magnificent dome of Pike's Peak; immediately in front, to the left, and about eight miles away, towers Cheyenne Mountain, and on the right is the beautiful Garden of the Gods. A hotel will be erected shortly at Colorado Springs, to cost, it is said, at least one hundred thousand dollars; and at the springs a bottling business will be established, with apparatus for bottling the waters and saving the carbonic gas.

